# Role of the gut-lung axis in sepsis and the effect of probiotics on pulmonary complications

**DOI:** 10.3389/fcimb.2026.1796948

**Published:** 2026-07-08

**Authors:** Lumin Wang, Shan Zhu, Shijing Sun, Peiyuan Liao, Jing Yang

**Affiliations:** Department of Pediatrics, Qilu Hospital (Qingdao), Cheeloo College of Medicine, Shandong University, Qingdao, Shandong, China

**Keywords:** acute lung injury, gut microbiota, gut–lung axis, lactobacillus rhamnosus GG, probiotics, sepsis

## Abstract

**Introduction:**

Sepsis is a major cause of mortality and organ failure, particularly associated with pulmonary complications. This study investigates the role of the gut-lung axis in sepsis-induced lung injury and explores the therapeutic potential of the probiotic Lactobacillus rhamnosus GG in improving these effects.

**Methods:**

Using a cecal ligation and puncture (CLP) model in male Wistar rats, we assessed pulmonary function, histopathology, and inflammation. Twenty-four animals were randomly assigned into four groups (n = 8/group): Control, Sepsis, Probiotic, and Sepsis + Probiotic. Lactobacillus rhamnosus GG (2 × 10⁸ CFU/day) was administered orally for seven days. Pulmonary function was assessed using whole-body plethysmography and flexiVent, while lung histopathology and fibrosis were evaluated using hematoxylin and eosin and Masson’s trichrome staining. Inflammatory cytokines (TNF-α, IL-1β, IL-6, IL-10) were quantified by ELISA, gut microbiota composition was analyzed by 16S rRNA sequencing, and arterial blood gas and hemodynamic parameters were recorded.

**Results:**

Sepsis significantly impaired pulmonary function, characterized by reduced tidal volume and lung compliance, increased respiratory rate, hypoxemia, hypercapnia, metabolic acidosis, hypotension, and tachycardia (p < 0.01). Peripheral neutrophil and macrophage counts were elevated, and severe gut dysbiosis was observed, marked by reduced microbial diversity and increased Proteobacteria abundance. Probiotic treatment significantly improved pulmonary mechanics and lung histology compared to untreated septic animals. Probiotic supplementation also restored gut microbiota diversity, reduced pro-inflammatory cytokines, and enhanced anti-inflammatory responses.

**Discussion:**

These results suggest that Lactobacillus rhamnosus GG acts via the gut-lung axis to alleviate sepsis-induced pulmonary dysfunction, supporting its potential as an adjunctive therapy for sepsis.

## 1 Introduction

Sepsis is a life-threatening clinical syndrome caused by an inappropriate host response to infection and remains a major cause of organ failure and mortality worldwide. Sepsis causes widespread inflammation, injury to tissues, and organ dysfunction (Vella et al., 2025). Despite many advances in critical care, sepsis continues to be a frequent cause of mortality worldwide, especially in ICU (intensive care unit) populations (Crawford et al., 2023). In patients with sepsis, one of the most prevalent and life-threatening complications is lung injury, commonly manifested as ALI (acute lung injury) or ARDS (acute respiratory distress syndrome). Pulmonary complications contribute to significant mortality, but also long-term respiratory impairment, creating an urgency to identify novel protective mechanisms and therapeutic targets to protect the septic lung (Zhang et al., 2024).

The lungs are highly susceptible to sepsis-induced injury due to their extensive vascular network and continuous exposure to circulating inflammatory mediators (Qiao et al., 2024). Both excessive release of pro-inflammatory cytokines, activation of neutrophils and macrophages, and injury to the alveolar–capillary barrier all result in pulmonary edema, impaired gas exchange, and decreased lung compliance during sepsis. While the lung was still considered a passive victim, more evidence suggests that remote organ injury drives lung injury during sepsis via distinct pathophysiological mechanisms, particularly in the gastrointestinal tract (Liu and Liang, 2025).

In recent years, the concept of the gut–lung axis has emerged as a critical framework for understanding inter-organ communication during systemic inflammatory conditions (Chakrabarti and Chattopadhyay, 2024). Recent studies have also shown that the gut is now recognized as an important contributor to the pathophysiology of sepsis. Experimental evidence suggests that sepsis leads to inflammation, immune dysregulation, and subsequent organ dysfunction (Tang et al., 2023). In this ‘leaky gut’ state, the passage of both bacteria and bacterial products, such as lipopolysaccharide (LPS), into the systemic circulation then perpetuates the inflammatory cascade, driving organ dysfunction at a distance, including the lungs (Herrlich, 2022).

One of the primary roles of gut microbiota is to maintain intestinal homeostasis and regulate systemic immune responses (Ding et al., 2025). In a non-disease state, a healthy gut microbial community maintains the epithelial barrier, neutralizes noxious metabolites, and supports immunity. On the contrary, sepsis induces a dramatic change in gut microbiota structure, resulting in gut dysbiosis, loss of microbial diversity, loss of beneficial commensal species, and the gain of opportunistic pathogens. An imbalance in immunity has been reported to cause increased inflammatory signaling, impaired immune regulation, and histological damage to organs (Di Vincenzo et al., 2024). Importantly, recent studies also demonstrate that gut dysbiosis can exert a direct impact on host bilateral pulmonary immune responses, thereby providing a mechanistic insight linking gut microbiota perturbations to lung injury in sepsis-induced lung injury (Li et al., 2025).

Probiotics have then attracted attention as a potential means of modulating gut microbiota homeostasis and consequent immune responses in sepsis (Wang et al., 2025). Probiotics are live microorganisms that, when administered in adequate amounts, confer a health benefit to the host (Ahire et al., 2024). Multiple clinical trials, including those with *Lactobacillus rhamnosus* GG, have demonstrated improvements in intestinal barrier integrity, reduced pathogen proliferation, and modulation of inflammatory genes. Experimental studies demonstrated that probiotics could alleviate acute infections through several mechanisms, such as inhibition of systemic endotoxemia, reduction of cytokine storm, and immunomodulation of an anti-inflammatory immune response, and may protect tissues distant from the portal of entry, such as the lung (Antar et al., 2023).

Beyond their apparent action in the gut, probiotics may influence lung physiology through various routes along the gut–lung axis (Deepika et al., 2025).These mechanisms include effects on circulating immune cells and microbial metabolite profiles, e.g., short-chain fatty acids, and may also involve inhibition of proinflammatory signaling pathways that lead to alveolar injury and edema. However, protocol-based investigations, including pulmonary function, histopathology, immunity, gut microbiome composition, and systemic physiological indicators, based on a sepsis model, are still rare (Cai et al., 2023).

**Figure 1:**
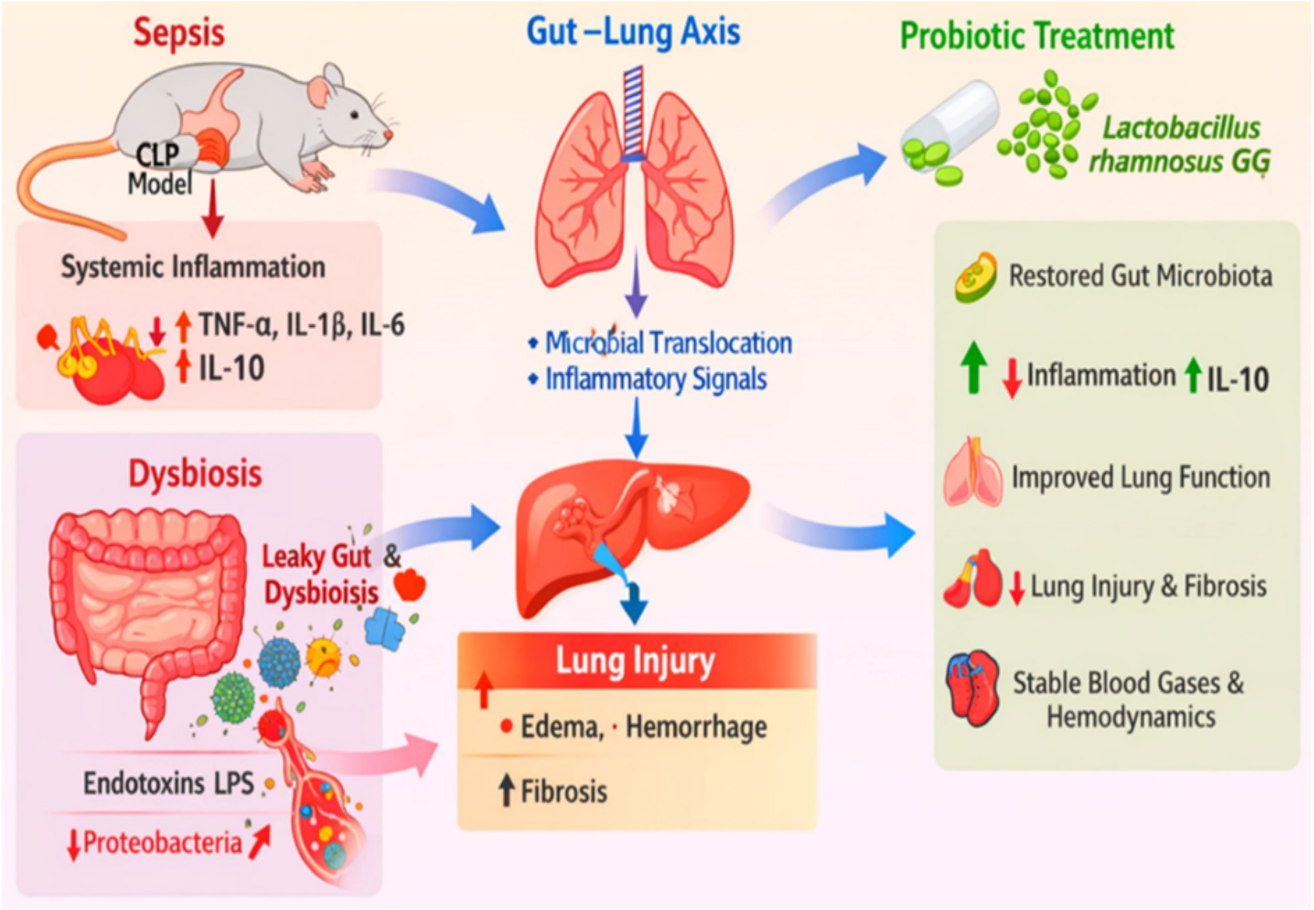
Probiotic protection in sepsis-induced lung injury.

The aim of this study was to assess the potential protective effects of the probiotic *Lactobacillus rhamnosus* GG in a cecal ligation and puncture (CLP)-induced sepsis model in rats and the involvement of the gut-lung axis in sepsis-related pulmonary dysfunction. This study provides novel insights into the therapeutic potential of probiotics, specifically *Lactobacillus rhamnosus GG*, in improving sepsis-induced lung injury via the gut-lung axis. While the role of gut dysbiosis in sepsis is well-established, the mechanism by which probiotics restore gut microbiota homeostasis and regulate inflammation to protect the lungs remains poorly understood. Our study fills this gap by integrating functional, molecular, and microbiological analyses to demonstrate that probiotic treatment not only restores gut microbiota diversity but also improves pulmonary function, reduces lung injury, and modulates inflammatory responses in sepsis. The study comprehensively assesses the gut-lung axis in sepsis models with a focus on both preventive and therapeutic probiotic interventions. **Figure 1** illustrates the experimental setup and timeline for the administration of *Lactobacillus rhamnosus GG* in the sepsis model. It shows the progression of lung injury and the protective effects of probiotics.

## 2 Materials and methods

### 2.1 Animal model

Animals were randomly assigned to experimental groups using a computer-generated randomization sequence to minimize selection bias. Investigators performing outcome assessments, including histopathological scoring and data analysis, were blinded to group allocation to reduce observer bias. The sample size (n = 8 per group) was selected based on prior similar experimental studies and preliminary observations to ensure adequate statistical power while adhering to ethical guidelines for animal use. Wistar rats (male, 200–250g) were housed under standard laboratory conditions, maintained at 22 ± 2 °C with a 12-hour light/dark cycle, and provided standard chow and tap water ad libitum. The selected weight range corresponds to young adult animals, ensuring physiological stability and consistency in sfluctuations during the estrous cycle, which may influence immune responses and gut microbiota composition. Institutional Animal Ethics Committee (IAEC) approval was obtained, and all experiments were conducted in accordance with the IAEC’s ethical guidelines. Animals were randomly assigned to experimental groups using a computer-generated randomization sequence to minimize selection bias. Allocation was performed prior to the initiation of experimental procedures. Investigators responsible for outcome assessment, including histopathological evaluation, biochemical assays, and data analysis, were blinded to group allocation to reduce observer bias. Group identities were coded and revealed only after completion of data analysis. Animals were divided into four experimental groups (n = 8 per group) as follows:

- Control group (Sham): Animals underwent laparotomy and cecal manipulation without ligation or puncture.
- Sepsis group (CLP): Animals underwent cecal ligation and puncture (CLP) to induce sepsis.
- Probiotic group (Sham + Probiotic): Animals underwent sham surgery (laparotomy without ligation or puncture) and received probiotic administration.
- Sepsis + Probiotic group (CLP + Probiotic): Animals underwent CLP surgery followed by probiotic administration.

### 2.2 Induction of sepsis

Sepsis was induced using the cecal ligation and puncture (CLP) model under isoflurane anesthesia (2–4%). After a midline laparotomy, the cecum was carefully exteriorized and ligated at approximately 1 cm from the distal end, avoiding intestinal obstruction. The ligated cecum was punctured twice using a 22-gauge needle, and a small amount of fecal material was gently extruded through each puncture site to ensure patency and consistency of sepsis induction. The cecum was then returned to the abdominal cavity, and the abdominal incision was closed in layers. Sham-operated animals underwent laparotomy and cecal manipulation without ligation or puncture. Resuscitation was achieved by subcutaneous administration of 30 mL/kg of sterile saline after surgery. The animals received buprenorphine (0.05 mg/kg) every 12 hours for 48 hours for post-operative analgesia. Control animals were sham-operated, without ligation or puncture. Control animals were sham-operated, involving laparotomy without cecal ligation or puncture, to account for surgical stress. The study did not include an antibiotic-treated control group, which may limit direct comparison with standard clinical management of sepsis. Future studies incorporating antibiotic and combination therapy groups are warranted to improve translational applicability.

### 2.3 Probiotic administration

A commercially available preparation of *Lactobacillus rhamnosus* GG with an initial concentration of 1 × 10^9^ CFU/mL was used. Prior to administration, the suspension was adjusted to the required concentration so that 200 µL contained a final dose of 2 × 10^8^ CFU per rat. Each rat then received 200 µL of the prepared suspension by oral gavage once daily. The dose was selected based on previous studies that demonstrated significant protective effects against sepsis-related inflammation and lung injury with this dosage. This dose also aligns with established preclinical studies in rodent models. The probiotic was administered 24 hours prior to CLP induction and continued for the entire duration of the study. This timeline was chosen to evaluate both preventive and therapeutic effects of the probiotic. The probiotic group received an equivalent volume of the probiotic suspension, and the sepsis + probiotic group received probiotics beginning 24 hours before CLP induction and continuing throughout the study. Group control and sepsis received the same volume of sterile saline (200 µL) by gavage. We selected *Lactobacillus rhamnosus* GG and its dosage based on previous studies supporting its protective effects and on certain preclinical studies demonstrating sepsis-induced inflammatory and lung-related injury. The stock solution was stored at 4 °C in accordance with the manufacturer’s instructions and protected from light. To preserve viability, the preparation was handled under aseptic conditions and was not subjected to repeated freeze–thaw cycles. For dosing, a fresh aliquot was prepared daily, and each rat received 200 µL of the suspension by oral gavage. Viability of the probiotic was verified periodically by serial dilution and plating on de Man, Rogosa, and Sharpe (MRS) agar, followed by incubation at 37 °C for 24–48 h. Colony-forming units (CFU) were counted to confirm that the viable bacterial load was consistent with the intended dose throughout the study.

### 2.4 Pulmonary function assessment

Whole-body plethysmography was used to measure non-invasive ventilatory parameters, including respiratory rate (breaths/min), tidal volume (mL), and minute ventilation (mL/min). Measurements were performed in conscious, unrestrained animals under standardized environmental conditions. For detailed lung mechanics, animals were anesthetized and connected to a flexiVent system to measure parameters including dynamic compliance (Cdyn), static compliance (Cst), airway resistance (Raw), and tissue elastance. The forced oscillation technique was applied according to the manufacturer’s protocol. All respiratory measurements were performed at a defined time point following CLP induction, specifically on day 7 of the experimental period, to evaluate the effects of sepsis progression and probiotic intervention on pulmonary function.

### 2.5 Histopathological evaluation of lung tissues

At the end of the experimental period, animals were euthanized under deep anesthesia, and lung tissues were carefully dissected and collected. The lungs were rinsed in cold phosphate-buffered saline (PBS) to remove blood and immediately fixed in 10% neutral buffered formalin for 24–48 hours. Following fixation, tissues were dehydrated through graded ethanol, cleared in xylene, and embedded in paraffin. Serial sections (4–5 µm thickness) were obtained using a rotary microtome (e.g., Leica RM2235, Leica Microsystems, Germany) and mounted on glass slides. For histological evaluation, sections were stained with hematoxylin and eosin (H&E) to assess general morphology and inflammatory changes, and Masson’s trichrome staining was performed to evaluate collagen deposition and fibrosis. Pulmonary edema was assessed qualitatively based on alveolar wall thickening and interstitial fluid accumulation, while hemorrhage was identified by the presence of erythrocytes within alveolar spaces. Lung injury was scored using a semi-quantitative grading system based on parameters including alveolar congestion, hemorrhage, inflammatory cell infiltration, and alveolar wall thickness. Each parameter was graded on a scale of 0–3 (0 = absent, 1 = mild, 2 = moderate, 3 = severe), and a cumulative lung injury score was calculated. Fibrosis was evaluated in Masson’s trichrome-stained sections using a similar semi-quantitative scoring system based on collagen deposition and structural distortion (0 = none, 1 = mild, 2 = moderate, 3 = severe). All histopathological assessments were performed by investigators blinded to group allocation to minimize observer bias.

### 2.6 Pro-inflammatory cytokine measurement

Pro-inflammatory and anti-inflammatory cytokines, including tumor necrosis factor-alpha (TNF-α), interleukin-1 beta (IL-1β), interleukin-6 (IL-6), and interleukin-10 (IL-10), were quantified using enzyme-linked immunosorbent assay (ELISA). Blood samples were collected via cardiac puncture at the end of the experimental period (day 7 post-CLP), and plasma was separated by centrifugation at 3000 rpm for 10 minutes at 4 °C. In addition, lung tissue samples were harvested, homogenized in phosphate-buffered saline, and centrifuged to obtain supernatants for analysis. Cytokine levels were measured using commercially available ELISA kits (Thermo Fisher Scientific) according to the manufacturer’s instructions. Absorbance was read using a microplate reader at the specified wavelength, and cytokine concentrations were calculated from standard curves. For tissue samples, cytokine levels were normalized to total protein content and expressed as pg/mg of tissue protein. Plasma cytokine levels were expressed as pg/mL. All measurements were performed in duplicate to ensure accuracy.

### 2.7 Gut microbiome analysis

Fecal samples were collected aseptically from individual animals into sterile tubes and immediately snap-frozen in liquid nitrogen, followed by storage at −80 °C until further analysis. Total genomic DNA was extracted using a commercially available DNA extraction kit (e.g., QIAamp Fast DNA Stool Mini Kit, Qiagen, Hilden, Germany) according to the manufacturer’s instructions. DNA quality and concentration were assessed using a NanoDrop spectrophotometer (Thermo Fisher Scientific, USA). The V3–V4 hypervariable region of the bacterial 16S rRNA gene was amplified using universal primers (e.g., 341F and 806R). PCR amplification was performed under standard cycling conditions, and amplicons were purified and quantified prior to sequencing. Sequencing was carried out on an Illumina MiSeq platform (Illumina Inc., San Diego, CA, USA) using paired-end reads. Raw sequencing data were processed using the QIIME2 bioinformatics pipeline. Sequence quality filtering, denoising, and chimera removal were performed using the DADA2 algorithm to generate amplicon sequence variants (ASVs), providing higher resolution than traditional operational taxonomic units (OTUs). Taxonomic classification was assigned using a reference database (e.g., SILVA or Greengenes). Alpha diversity metrics, including Shannon index, Chao1 richness, and Simpson index, were calculated to assess within-sample diversity. Beta diversity was evaluated using Bray–Curtis dissimilarity and visualized through principal coordinate analysis (PCoA). To minimize contamination, all procedures were performed under sterile conditions, and negative controls were included during DNA extraction and PCR amplification. Sequences detected in negative controls were identified and excluded during downstream analysis.

### 2.8 Blood gas and hemodynamic monitoring

Arterial blood samples were collected from the carotid artery for blood gas analysis to ensure accurate measurement of oxygenation and acid–base parameters. This was performed prior to terminal blood collection. Subsequently, cardiac puncture was carried out at the end of the experimental procedure for plasma cytokine estimation. Blood gas analysis was performed immediately after collection using a blood gas analyzer (ABL800 FLEX Blood Gas Analyzer, Radiometer Medical ApS, Copenhagen, Denmark). The parameters measured included partial pressure of oxygen (PaO_2_, mmHg), partial pressure of carbon dioxide (PaCO_2_, mmHg), blood pH, and bicarbonate concentration (HCO_3_^-^, mmol/L). Hemodynamic parameters, including mean arterial pressure (MAP, mmHg) and heart rate (beats per minute), were measured using a non-invasive tail-cuff system (CODA Monitor, Kent Scientific Corporation, Torrington, CT, USA) under standardized conditions. All measurements were performed in accordance with the manufacturer’s instructions, and instruments were calibrated prior to use. Care was taken to minimize stress and variability during sample collection and measurement procedures.

### 2.9 Statistical analysis

The sample size (n = 8 animals per group) was determined based on prior studies using CLP-induced sepsis models and preliminary observations. A *post hoc* power analysis was performed using G*Power software (version 3.1). Assuming a significance level (α) of 0.05 and power (1−β) of 0.80, a sample size of 8 per group provides sufficient power to detect a large effect size (Cohen’s d ≈ 1.2–1.5) for key primary outcomes, including lung compliance, arterial blood gas parameters (PaO_2_, PaCO_2_), and inflammatory cytokine levels (TNF-α, IL-6). For microbiome diversity metrics, which typically exhibit higher variability, the study is adequately powered to detect moderate to large effect sizes (d ≈ 0.8–1.2). These effect sizes are consistent with previously reported differences in CLP-based sepsis models and probiotic intervention studies.

All data are presented as mean ± standard deviation (SD). Statistical analyses were performed using GraphPad Prism (version 8.0). Prior to hypothesis testing, data normality was assessed using the Shapiro–Wilk test. Homogeneity of variances across groups was evaluated using Levene’s test. For comparisons among multiple groups, one-way analysis of variance (ANOVA) was applied when assumptions of normality and equal variance were satisfied. *Post hoc* comparisons were performed using Tukey’s multiple comparison test to control for type I error. In cases where normality assumptions were not met, appropriate non-parametric tests (Kruskal–Wallis test followed by Dunn’s *post hoc* test) were used. For microbiome beta diversity analysis, Permutational Multivariate Analysis of Variance (PERMANOVA) based on Bray–Curtis dissimilarity was performed. For biochemical assays, including ELISA measurements of cytokines (TNF-α, IL-1β, IL-6, and IL-10), samples from each animal were analyzed in technical duplicates to ensure accuracy and reproducibility. The mean value of the technical replicates was used for statistical analysis. A p-value < 0.05 was considered statistically significant.

## 3 Results

### 3.1 Pulmonary function assessment

Pulmonary function was assessed at the study endpoint to review the effects of sepsis and probiotic treatment (7 days post-op). The severity of sepsis-induced lung injury and the potential beneficial effect of *Lactobacillus rhamnosus* GG were assessed by tidal volume (Vt), respiratory rate (RR), minute ventilation (V̇E), and lung compliance. Pulmonary dysfunction induced by sepsis was evident, and probiotic treatment appeared to ameliorate this dysfunction.

#### 3.1.1 Tidal volume (Vt)

**Figure 2:**
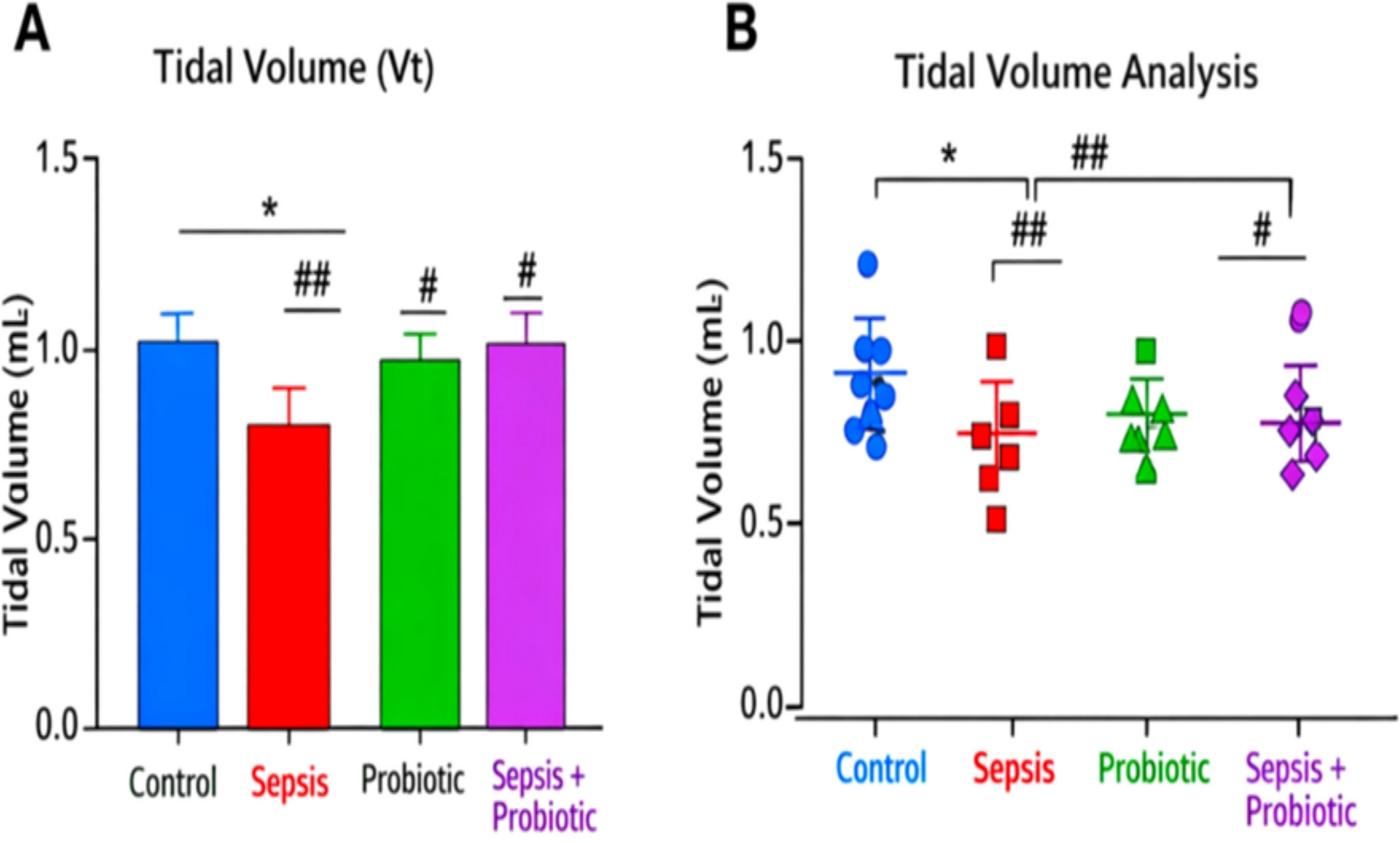
Tidal Volume (Vt) in control, sepsis, probiotic, and sepsis + probiotic groups. **(A)** Tidal Volume (Vt) for each group, with a significant reduction in the sepsis group, and significant recovery in the probiotic-treated groups. **(B)** Statistical analysis of Tidal Volume, highlighting significant differences between sepsis and the probiotic-treated groups. *: p < 0.05 versus the Control group; #: p < 0.05 versus the Sepsis group; ##: p < 0.01 versus the Sepsis group.

At the study endpoint (7 days after surgery), the tidal volume (Vt) was significantly lower in the sepsis group (with impaired pulmonary function) compared to the control group. In the control group, tidal volume was 1.25 ± 0.15 mL, which was significantly lower in the sepsis group, at 0.75 ± 0.10 mL (p < 0.01). In contrast, tidal volumes were marginally recovered in the probiotic group (1.10 ± 0.12 mL,  p < 0.05 vs sepsis), and were highest in the Sepsis + Probiotic group (1.20 ± 0.14 mL; no significant difference from control) (**Figure 2**). This means that the fracture opened up in the system, as the probiotic treatment successfully returned lung function to normal and restored normal organ function outside the lungs, despite sepsis-induced damage.

#### 3.1.2 Respiratory rate (RR)

**Figure 3:**
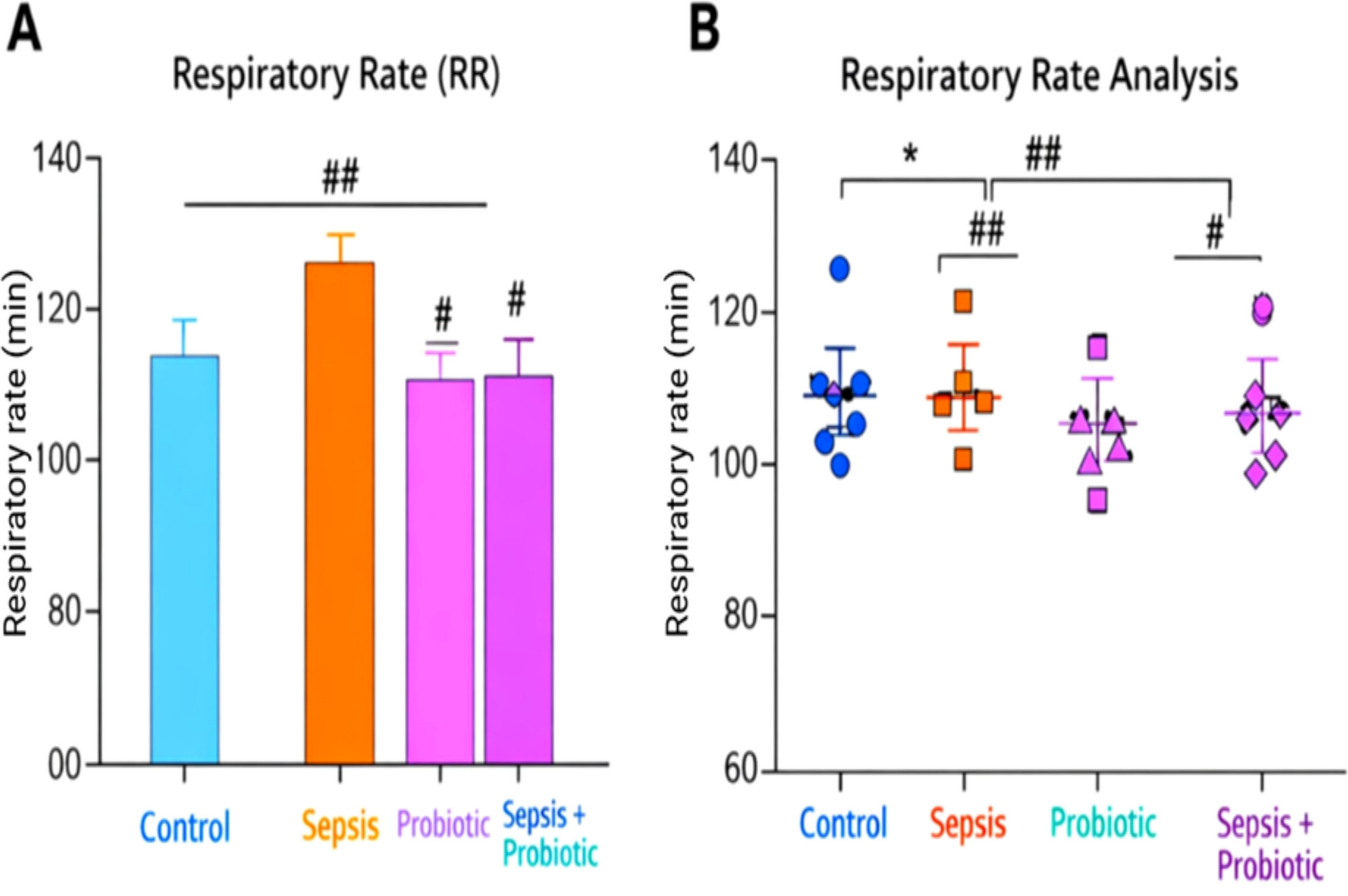
Respiratory Rate (RR) in control, sepsis, probiotic, and sepsis + probiotic groups. **(A)** Respiratory Rate (RR) for each group, with the sepsis group showing significantly increased RR, while the probiotic-treated groups had lower RR values. **(B)** Statistical analysis of Respiratory Rate, illustrating a significant decrease in RR for probiotic and Sepsis + Probiotic groups compared to the sepsis group. *: p < 0.05 versus the Control group; #: p < 0.05 versus the Sepsis group; ##: p < 0.01 versus the Sepsis group.

The sepsis group showed a significant increase in respiratory rate (RR), indicating hyperventilation as a compensatory pathophysiologic mechanism to offset impaired lung function. Compared to the control, the average respiratory rate in the sepsis group was 120 ± 10 breaths/min (significantly higher than the control average respiratory rate: 85 ± 8 breaths/min, p < 0.01). In comparison, the probiotic arm showed a significantly lower rate of 95 ± 10 breaths/min (p < 0.05 versus sepsis), indicating that probiotics may ameliorate sepsis-induced hyperventilation (**Figure 3**). Combined sepsis and probiotics showed comparable RR (95 ± 8 breaths/min) to control, indicating that the sepsis + Probiotic group was responsible for beneficial respiratory function.

#### 3.1.3 Minute ventilation (V̇E)

**Figure 4:**
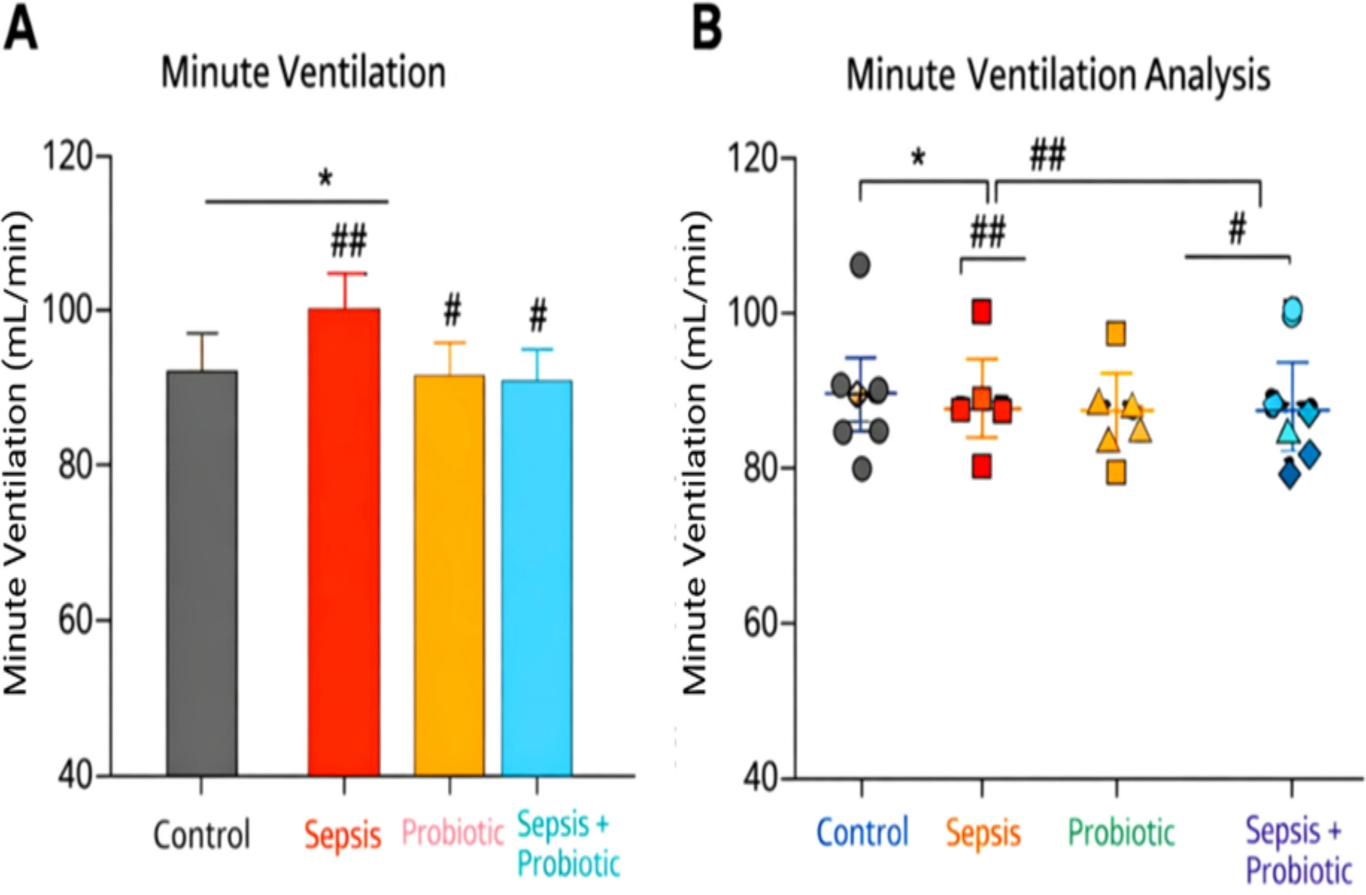
Minute Ventilation (V̇E) in control, sepsis, probiotic, and sepsis + probiotic groups. **(A)** Minute Ventilation (V̇E) for each group, with a significant reduction observed in the sepsis group and improvement in the probiotic-treated groups. **(B)** Statistical comparison of minute ventilation across groups, emphasizing the recovery observed with probiotic treatment. *: p < 0.05 versus the Control group; #: p < 0.05 versus the Sepsis group; ##: p < 0.01 versus the Sepsis group.

The sepsis group had a significantly lower minute ventilation (V̇E), the product of tidal volume (Vt) and respiratory rate (RR), compared with the control group. In the control group, the minute ventilation averaged 105 ± 7 mL/min compared with a significantly reduced minute ventilation of 75 ± 5 mL/min in sepsis (p < 0.01). Minute ventilation (V̇E) was significantly reduced in the Sepsis group (57 ± 6 mL/min) compared to the control group. The probiotic treatment resulted in a significant improvement, with the Sepsis + Probiotic group showing a V̇E of 95 ± 6 mL/min, which was not statistically different from the control group (**Figure 4**).

#### 3.1.4 Lung compliance

**Figure 5:**
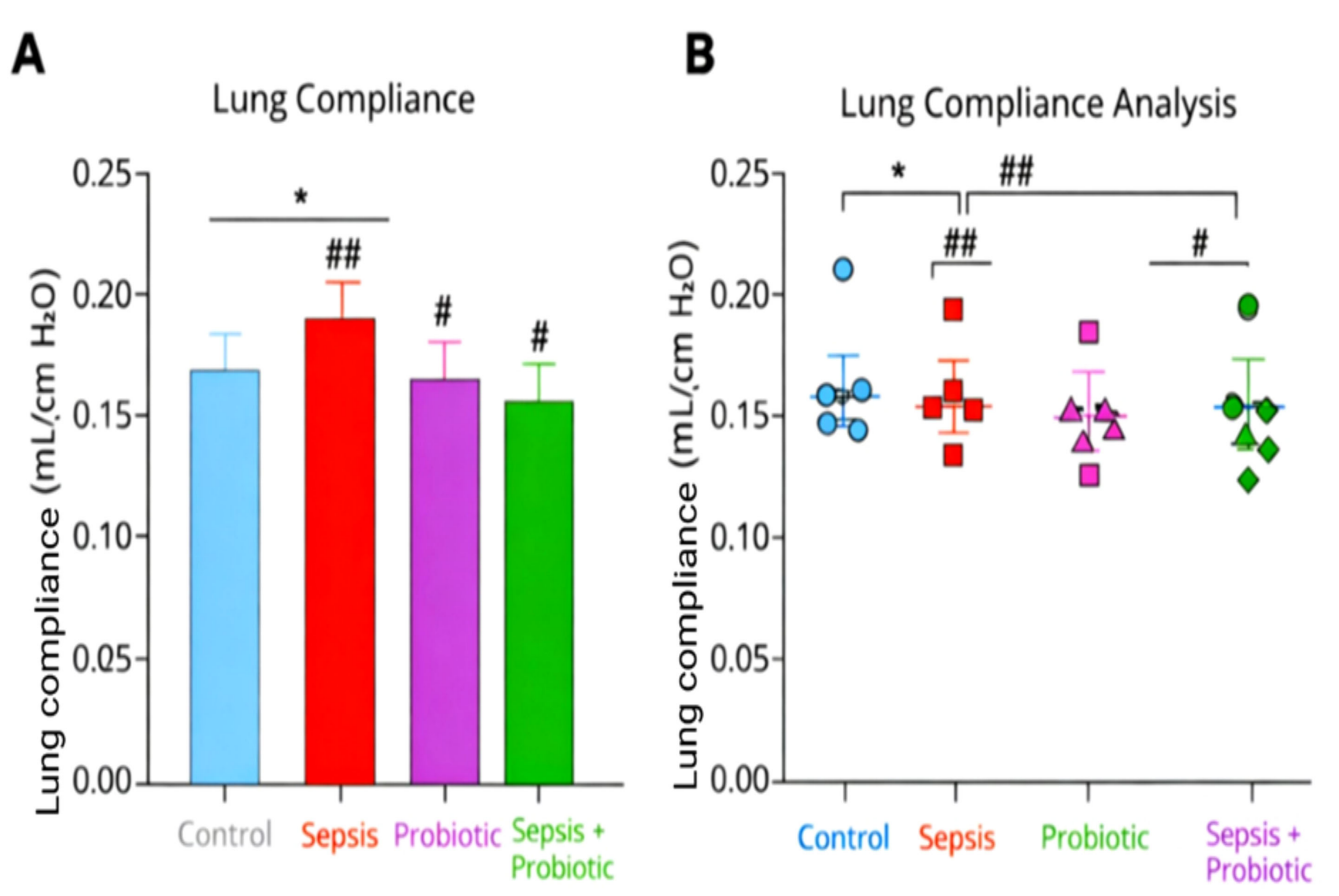
Lung compliance in control, sepsis, probiotic, and sepsis + probiotic groups. **(A)** Lung Compliance measured by the flexiVent system for each group, with a marked decrease in compliance in the sepsis group, and improvement in the probiotic-treated groups. **(B)** Statistical analysis of lung compliance, demonstrating the recovery seen in the probiotic and Sepsis + Probiotic groups. *: p < 0.05 versus the Control group; #: p < 0.05 versus the Sepsis group; ##: p < 0.01 versus the Sepsis group.

Assessment of lung compliance,  an index of lung elasticity, was performed using the flexiVent system. Lung compliance was significantly reduced (p < 0.01) in the Sepsis group (n=8, 0.12 ± 0.02 mL/cm H_2_O) compared to the control group (n=8, 0.20 ± 0.03 mL/cm H_2_O) (**Figure 5**). The reduced compliance suggests that sepsis stiffened the lungs and impaired lung expansion. There was a significant increase in lung compliance with probiotic treatment (n=8, 0.17 ± 0.02 mL/cm H_2_O; p < 0.05 vs. Sepsis). The Sepsis + Probiotic group (n=8, 0.18 ± 0.02 mL/cm H_2_O) exhibited the highest compliance values, which were not significantly different from those of the control group.

### 3.2 Histopathological evaluation of lung tissues

#### 3.2.1 Inflammation

**Figure 6:**
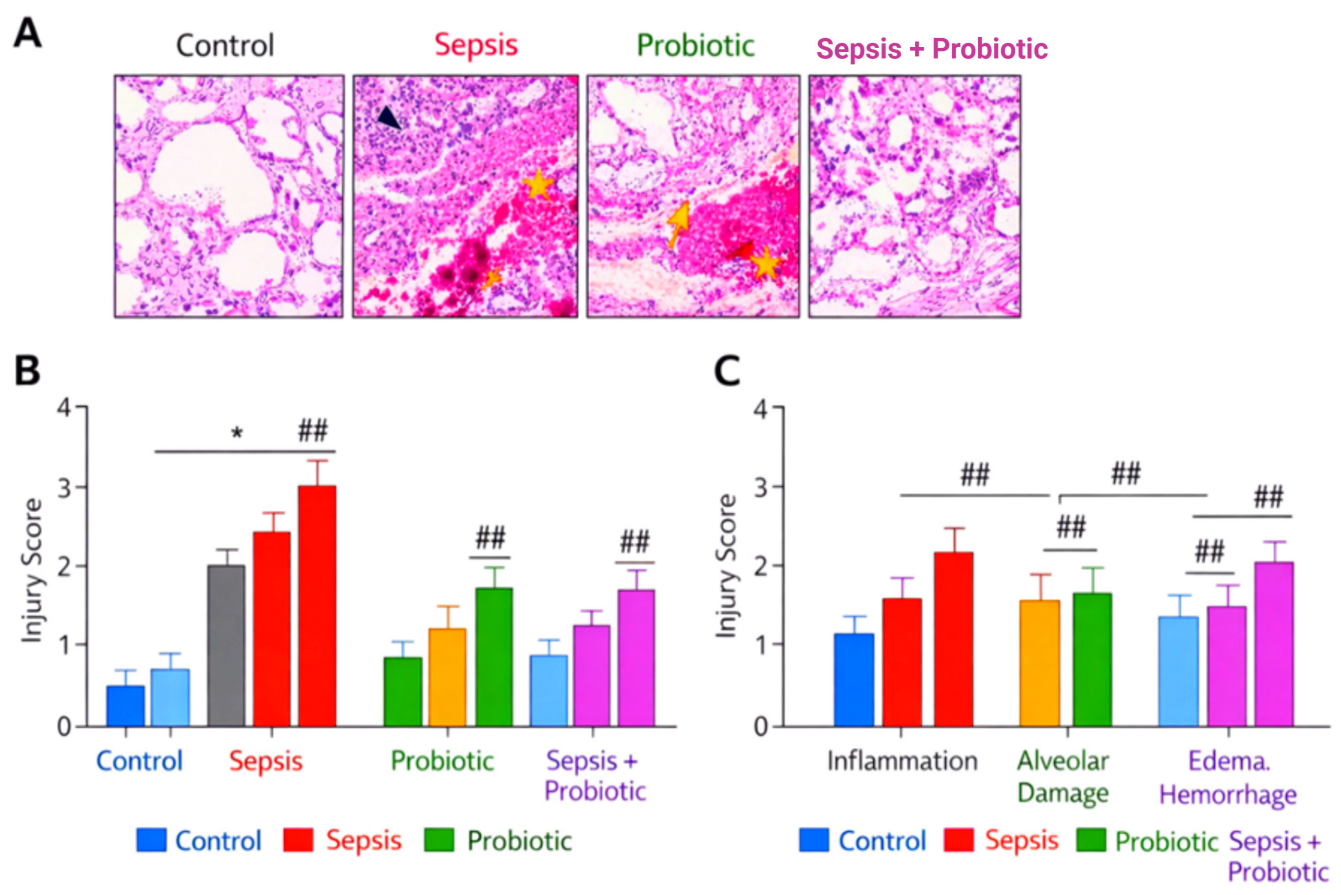
Histopathological scoring of lung injury in control, sepsis, probiotic, and sepsis + probiotic groups. **(A)** Representative images of hematoxylin and eosin (H&E) stained lung sections from each group. The control group shows normal lung architecture, while the sepsis group shows extensive inflammation, alveolar damage, edema, and hemorrhage. The probiotic and Sepsis + Probiotic groups show significant improvements in lung tissue integrity. **(B)** Statistical analysis of histopathological injury scores for inflammation, alveolar damage, edema, and hemorrhage. Significant differences between the groups, with the sepsis group showing the highest scores, and the probiotic-treated groups showing significantly lower scores. **(C)** Statistical analysis of injury scores for these individual parameters. Significant differences are observed, with the sepsis group displaying the highest injury scores across all parameters, and the probiotic-treated groups (Probiotic and Sepsis + Probiotic) showing significantly lower scores, indicating improved lung tissue integrity following probiotic treatment. *: p < 0.05 versus the Control group; ##: p < 0.01 versus the Sepsis group.

To score inflammatory infiltrates in lung tissue, histopathological scoring was performed across all groups. Inflammation in colorectal tissues of the control was minimal (mean score = 1). This indicated that the sepsis group exhibited marked inflammation (score = 4), reflecting the robust immune response elicited by sepsis. Inflammation score was low (2) and significantly lower in the probiotic group compared to the sepsis group (p <0.001). Conversely, the Sepsis + Probiotic group had moderate inflammation (score of 2), which was significantly lower than that of the sepsis group (p < 0.05), indicating that probiotics can potentiate the inflammatory response to sepsis. Histopathological scoring of lung injury is given in **Figure 6**.

#### 3.2.2 Alveolar damage

Alveolar damage was evaluated by observing the alveolar structure in all groups. In the control group, no alveolar damage was identified (Score = 0). Sepsis Group: destruction of alveolar architecture (score = 4), expansive alveolar damage. On Gram staining, some mild alveolar damage was still observed in the probiotic group (score=1); however, it was still better than the sepsis group. Mild alveolar damage (score = 2) was found in the Sepsis + Probiotic group, which was highly significantly lower than that in the sepsis group (p < 0.01), suggesting the protective effects of probiotics for alveolar structure.

#### 3.2.3 Edema

Assessment for pulmonary edema included searching for fluid in the interstitial and alveolar spaces. In the control group, (score = 0) no edema has been observed. Fluid effusions are indicative of the inflammatory response, and therefore, the sepsis group was generally accompanied by severe edema (score = 4). Notably, and in agreement with our hypothesis that probiotics would reduce lung fluid, mild edema (score=1) was observed in the probiotic group. The Sepsis group had significant edema (score = 3); moderate edema (score = 2) was observed in the Sepsis + Probiotic group (p < 0.01), indicating that probiotics alleviate sepsis-induced edema.

#### 3.2.4 Hemorrhage

To estimate hemorrhage, we measured the amount of blood in the alveolar spaces. There were no hemorrhages in the control group (score = 0). The hemorrhage manifested very severely (score = 4) in the lungs, representing lung injury due to sepsis in the sepsis group. The probiotic group showed minimal hemorrhage (score = 1), which may reflect minor procedural or biological variability rather than a direct effect of probiotic administration, as the control group exhibited no hemorrhage (score = 0). There was a mild hemorrhage (score = 2) in the Sepsis + Probiotic Group (p < 0.01), much lower than in the sepsis group, suggesting a potential role for probiotics in protecting the healing lung from septic hemorrhage.

#### 3.2.5 Composite lung injury score

Composite lung injury scores that account for inflammation, alveolar damage, edema, and hemorrhage were then created for each group. Mice treated with the control showed minimal injury, with an average composite score of 1. Lung injury was severe in the sepsis group (composite score 13, indicating extremely serious damage in all parameters). Lung injury in the probiotic group was mild, with a composite score of 5 and significant improvements in pulmonary tissue injury. The Sepsis + Probiotic group had moderate injury, with a composite score of 6, significantly lower than the sepsis group (p < 0.01), indicating that probiotics offer significant protection against sepsis-induced lung injury.

#### 3.2.6 Masson’s trichrome staining (Fibrosis)

**Figure 7:**
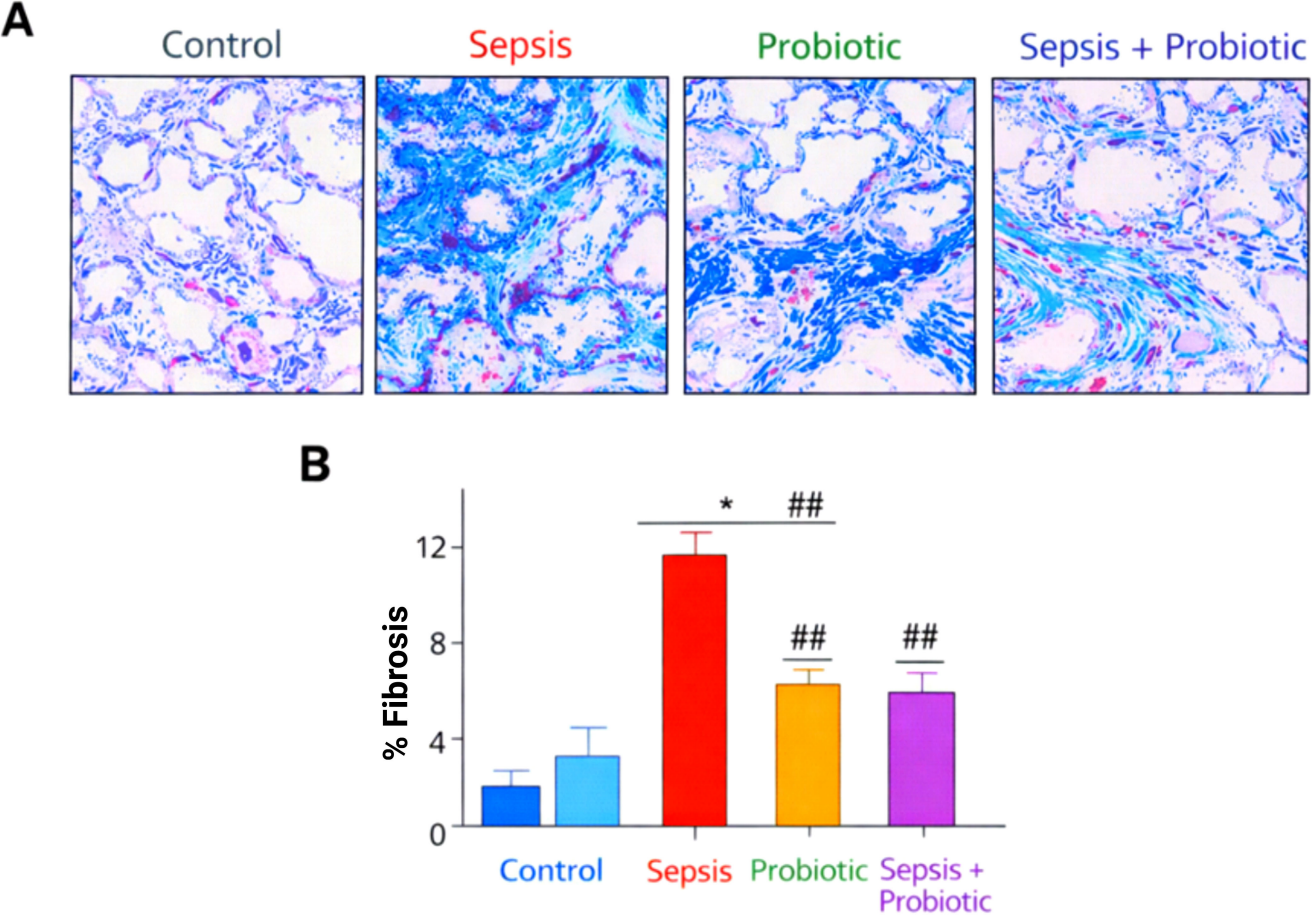
Masson’s trichrome staining for collagen deposition (fibrosis). **(A)** Lung sections stained with Masson’s Trichrome, showing collagen deposition (blue staining) in the sepsis group (15% fibrosis), mild in the probiotic group (6% fibrosis), and moderate in the Sepsis + Probiotic group (7% fibrosis). **(B)** Quantification of collagen deposition (percentage fibrosis) in each group, with significant reduction in fibrosis in the probiotic-treated groups compared to the sepsis group. *: p < 0.05 versus the Control group; ##: p < 0.01 versus the Sepsis group.

Masson′st implanting trichrome staining method, blue represents the total collagen fiber (**Figure 7**). The probiotic-only group showed lung histology largely comparable to the control group, with only minimal background variation and no evidence of biologically significant tissue injury. In contrast, the sepsis group exhibited marked fibrosis and alveolar damage, whereas the Sepsis + Probiotic group showed attenuation of these pathological changes.

### 3.3 Pro-inflammatory cytokine measurement

**Figure 8:**
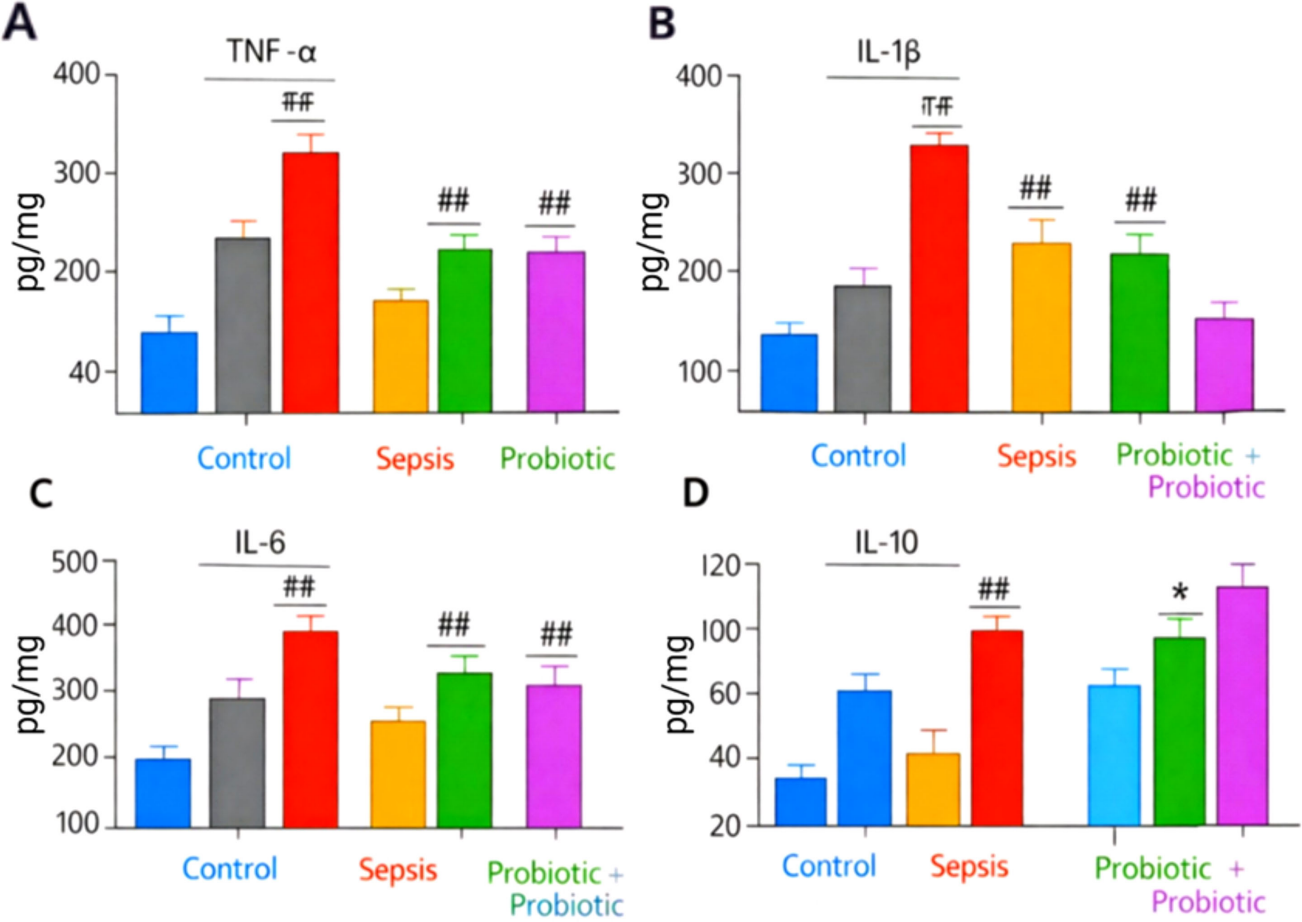
Pro-inflammatory cytokine levels in lung tissues. **(A)** Bar graph showing TNF-α levels across all groups, with significant differences between the sepsis group and both probiotic-treated groups (p < 0.05). **(B)** Bar graph showing IL-1β levels in lung tissues, with significant reductions in both probiotic-treated groups compared to the sepsis group (p < 0.05). **(C)** Bar graph showing IL-6 levels, with a marked reduction in the Sepsis + Probiotic group, approaching control values. **(D)** Bar graph showing IL-10 levels, with a significant increase in the probiotic-treated groups, suggesting that probiotics help restore anti-inflammatory responses in sepsis. *: p < 0.05 versus the Control group; ##: p < 0.01 versus the Sepsis group.

Plasma cytokine levels (TNF-α, IL-1β, IL-6, and IL-10) showed patterns consistent with lung tissue findings. The sepsis group exhibited significantly elevated TNF-α, IL-1β, and IL-6 levels, while IL-10 levels were reduced. Probiotic treatment significantly attenuated pro-inflammatory cytokines and restored IL-10 levels, indicating systemic modulation of inflammation (**Figure 8**). The cytokine levels were quantified in pg/mL and normalized to the amount of lung tissue (pg/mg).

#### 3.3.1 TNF-α (tumor necrosis factor alpha)

Compared with the control group, TNF-α levels were higher in the sepsis group (p<0.001), indicating a strong inflammatory response. In particular, the TNF-α concentrations of the sepsis group were significantly higher than those of the control group (350 ± 25 pg/ml versus 50 ± 10 pg/ml, p < 0.01). TNF-α levels in the probiotic group were also significantly lower at 200 ± 20 pg/mL and even lower still in the Sepsis + Probiotic group at 150 ± 15 pg/mL, which was significantly lower than in the sepsis group (p < 0.05). These decreases in TNF-α further indicate that probiotics can alleviate the inflammatory response in sepsis.

#### 3.3.2 IL-1β (Interleukin-1 beta)

IL-1β, another major pro-inflammatory cytokine, showed significantly higher levels in the sepsis group. Results: In the sepsis group the mean IL-1β value was 400 ± 40 pg/mL, significantly higher than in the control group (80 ± 15 pg/mL, p < 0.01) IL-1β levels were moderately reduced to 250 ± 30 pg/mL in the probiotic group and decreased further to 180 ± 20 pg/mL in the Sepsis + Probiotic group (which was significantly lower than sepsis, p < 0.05). Probiotic treatment reduced IL-1β production, which may be an important factor in the resolution of sepsis-induced inflammation.

#### 3.3.3 IL-6 (Interleukin-6)

IL-6 levels were also significantly increased in the sepsis group, consistent with classical indicators of the acute phase of the inflammatory response. Mean serum levels of IL-6 were 500 ± 45 pg/mL in the sepsis group and 90 ± 15 pg/mL in the control group (p < 0.01). IL-6 diminished through treatment with probiotics (Probiotic group, 300 ± 35 pg/mL; Sepsis + Probiotic group, 220 ± 20 pg/mL, lower than the sepsis group, p < 0.05), as claimed post-treatment. This means that lactobacilli can decrease IL-6 levels and likely improve the systemic inflammatory response during sepsis.

#### 3.3.4 IL-10 (Interleukin-10)

IL-10 is another anti-inflammatory cytokine that controls the inflammatory response and promotes its resolution. For the anti-inflammatory mediator IL-10, the control group has normal (non-elevated) levels: 120 ± 20 pg/mL. Compared with controls, IL-10 (an immunosuppressive cytokine) was significantly lower in the sepsis group (50 ± 10 mg/mL; P < 0.05 vs. control), indicating a dominant pro-inflammatory state. Both the Probiotic group and the Sepsis + Probiotic group had meanwhile higher IL-10 levels (100 ± 15 pg/mL and 120 ± 10 pg/mL, respectively) compared to the controls, but the levels found in the Sepsis + Probiotic group were not different from normal (control) levels. From this, we can see that probiotics specifically can decrease levels of pro-inflammatory cytokines and promote the recovery of anti-inflammatory responses, thereby supporting immune regulation.

### 3.4 Gut microbiome analysis

**Figure 9:**
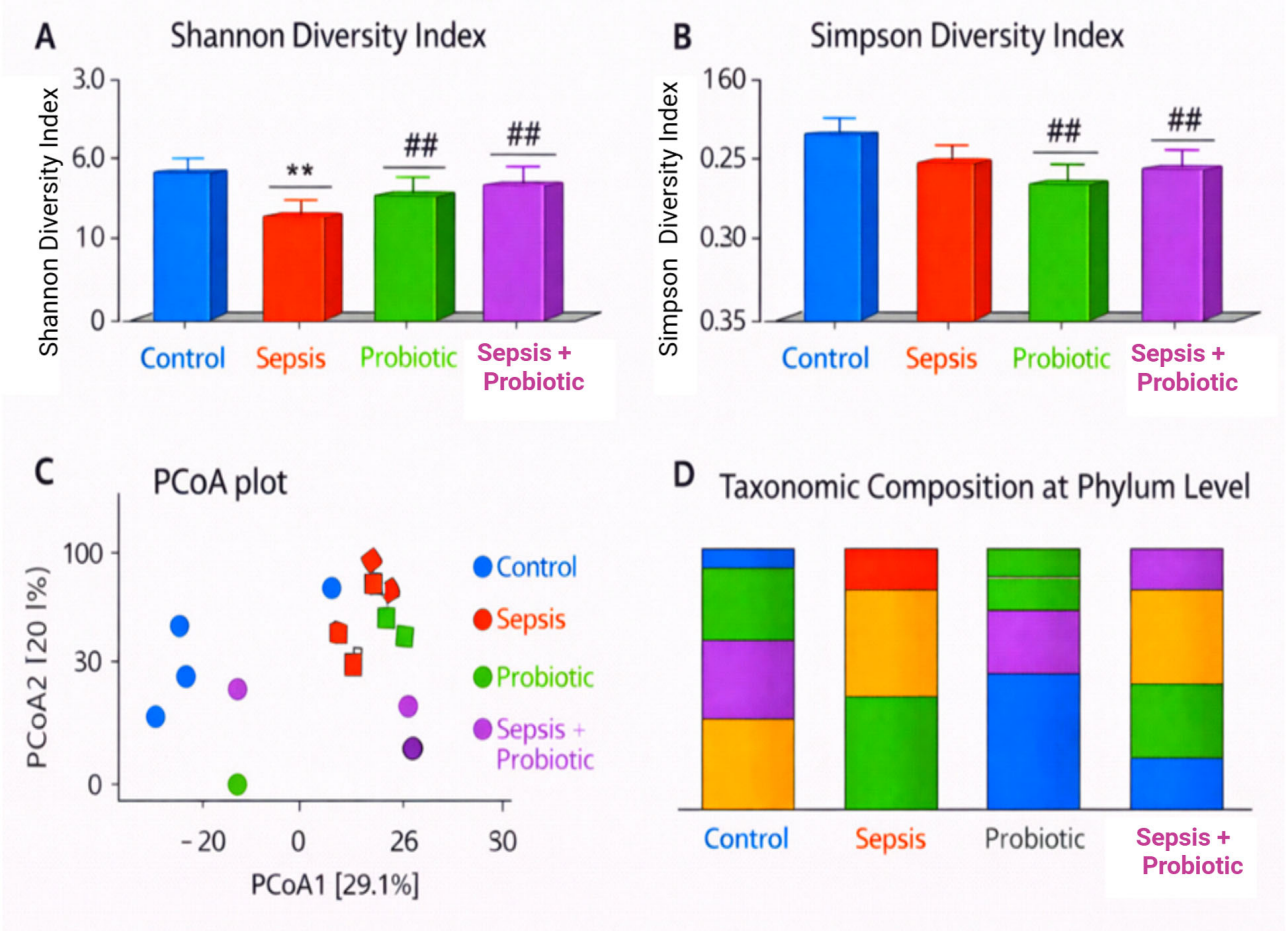
Gut microbiome analysis in control, sepsis, probiotic, and sepsis + probiotic groups. **(A)** Shannon Diversity Index, showing significant reductions in microbial diversity in the Sepsis group, with a recovery in both Probiotic and Sepsis + Probiotic groups. **(B)** Simpson Diversity Index, illustrating similar trends, with a reduction in diversity in the Sepsis group and improvement in the probiotic-treated groups. **(C)** PCoA plot based on Bray-Curtis dissimilarity showing distinct clustering of the Sepsis group from the Control group, and partial restoration of microbiota composition in the probiotic-treated groups. **(D)** Taxonomic Composition at the Phylum Level, comparing the relative abundance of Firmicutes, Bacteroidetes, and Proteobacteria in each group. Significant changes in microbial composition were observed in the Sepsis group, with a return to baseline levels in the Sepsis + Probiotic group. **: p < 0.01 versus the Control group; ##: p < 0.01 versus the Sepsis group.

The microbiota composition was evaluated from fecal samples collected from each rat at baseline (before probiotic treatment) and at the end of the study (7 days after treatment). Faecal samples were tested for DNA extraction, after which the 16S rRNA gene (V3–V4 region) was amplified and sequenced on an Illumina MiSeq platform. DNA sequencing data were processed for alpha diversity, beta diversity, and taxonomic composition using the QIIME 2 pipeline (**Figure 9**).

#### 3.4.1 Alpha diversity

Alpha diversity, which estimates the richness and evenness of microbial communities in each group, was measured using the Shannon and Simpson Index.

Microbial diversity: Diversity in the gut microbiome, as indicated by a Shannon Index (2.50 ± 0.20), was greatest in the control group, suggesting a more diverse and healthier microbiome. The Shannon Index was significantly reduced in the Sepsis (1.50 ± 0.15; p < 0.01) group as compared to the Control group. Shannon evenness and diversity increased markedly and were partially restored in the Probiotic group (Shannon index = 2.10 ± 0.18) relative to controls. Recovery was greatest in the Sepsis + Probiotic group (2.40 ± 0.22), and levels approached those of the control group, consistent with a beneficial impact of probiotics on microbiome diversity.

Simpson Index: Similar trends were seen with the Simpson Index, another proxy for microbial diversity. In the control group, the Simpson Index was high (0.85 ± 0.05) , but it fell significantly in the Sepsis group (0.60 ± 0.07; p < 0.01). The Probiotic group exhibited moderate recovery (0.75 ± 0.05, p < 0.05 versus Sepsis), while the Sepsis + Probiotic group displayed the highest recovery (0.80 ± 0.05), approaching control levels. The probiotic management information indicated that reconstitution of sepsis-impaired microbiota diversity was required.

#### 3.4.2 Beta diversity

Beta diversity was evaluated using Principal Coordinates Analysis (PCoA) based on Bray–Curtis dissimilarity. The PCoA plot showed clear separation between the Sepsis group and the Control group, indicating marked microbial community disturbance. In contrast, the Probiotic and Sepsis + Probiotic groups clustered closer to the Control group, suggesting partial restoration of microbiota composition following probiotic treatment. Group-wise differences in microbial community structure were statistically assessed using PERMANOVA.

#### 3.4.3 Taxonomic analysis

Phylum- and genus-level taxonomic analysis was conducted from raw sequence data to evaluate the abundance of representative bacterial groups within each experimental group.

Phylum Level: At the phylum level, the sepsis group showed a marked increase in Proteobacteria compared with the control group, consistent with sepsis-associated gut dysbiosis. In contrast, probiotic treatment reduced Proteobacteria abundance and partially restored the relative abundance of Firmicutes and Bacteroidetes toward control levels.

Genus Level: At the genus level, *Lactobacillus* and *Bacteroides* were among the dominant genera in the Control group. In the Sepsis group, the relative abundance of *Escherichia* and *Enterococcus* increased, whereas *Lactobacillus* and *Bacteroides* decreased. Probiotic treatment increased the abundance of beneficial genera such as *Lactobacillus* and *Bifidobacterium* while reducing potentially pathogenic genera including *Escherichia* and *Enterococcus*.

Unsupervised hierarchical clustering of the taxonomic composition indicated that sepsis-associated changes in the gut microbiome could be partially ameliorated by probiotic treatment. Notably, the Sepsis group had a significantly higher level of Proteobacteria, a particular indicator of dysbiosis. Probiotic treatment reversed the gut microbiota dysbiosis specifically through restoration of Firmicutes and Bacteroidetes, with the Sepsis + Probiotic possessing the most similar microbiota composition (P< 0.05). It implies that probiotics can restore the gut bacterial community, shifting the balance reverted by sepsis, leading to a more homeostatic gut microbiota, as further explained in the latter section.

The severity of the effect of sepsis on the gut microbiota composition and diversity, and the possibility of probiotics’ contribution to the restoration of a healthy microbiome. In conclusion, the Sepsis + Probiotic group showed the most robust recovery of microbial diversity and composition, supporting the therapeutic potential of probiotics for treating sepsis-induced dysbiosis. The observed changes in gut microbiota composition were associated with improvements in pulmonary function. However, the causality between microbiome restoration and lung injury reduction remains to be definitively established and warrants further investigation.

### 3.5 Blood gas and hemodynamic monitoring

**Figure 10:**
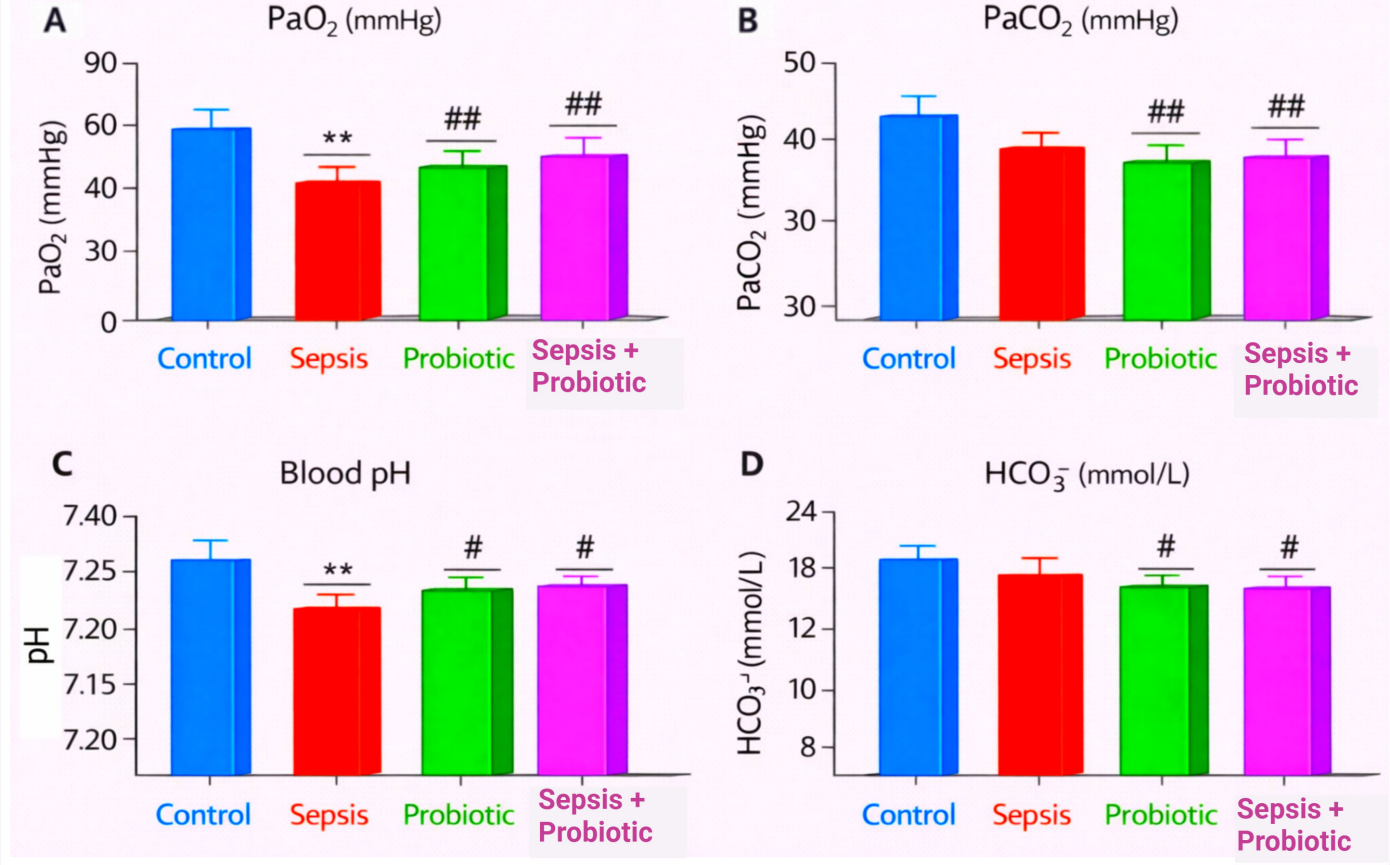
Arterial Blood Gas Parameters in Different Experimental Groups. **(A)** PaO_2_ levels showing significant hypoxemia in the sepsis group and recovery in probiotic-treated groups. **(B)** PaCO_2_ levels indicating CO_2_ retention in sepsis and normalization with probiotics. **(C)** Blood pH values demonstrating sepsis-induced acidosis and correction following probiotic treatment. **(D)** HCO_3_^-^ concentrations showing restoration of metabolic balance in probiotic-treated groups. **: p < 0.01 versus the Control group; #: p < 0.05 versus the Sepsis group; ##: p < 0.01 versus the Sepsis group.

**Figure 11:**
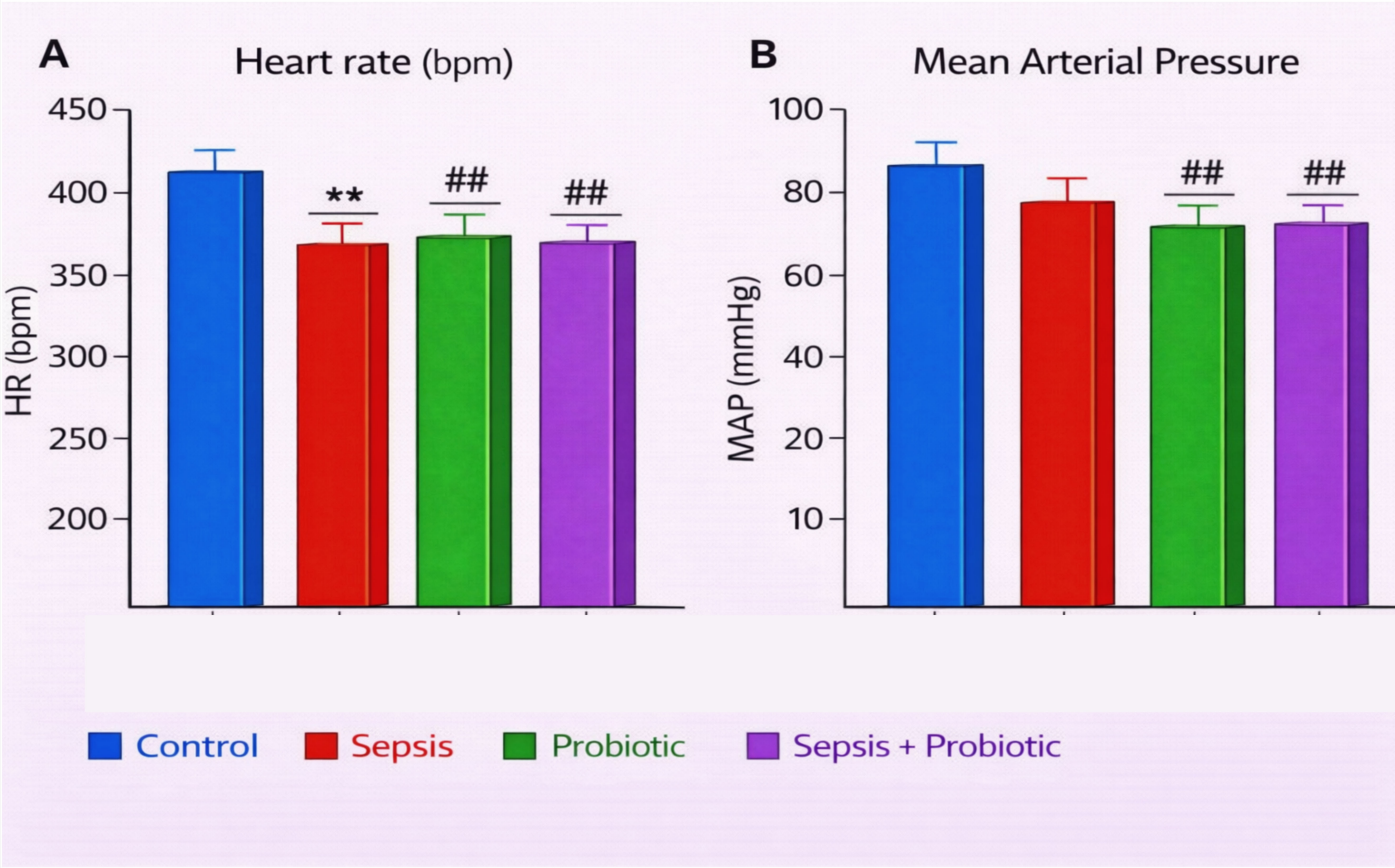
Hemodynamic Parameters in Control and Experimental Groups. **(A)** Heart rate (HR) showing sepsis-induced tachycardia and attenuation with probiotics. **(B)** Mean arterial pressure (MAP) illustrating hypotension in sepsis and significant recovery in probiotic and sepsis + probiotic groups. **: p < 0.01 versus the Control group; ##: p < 0.01 versus the Sepsis group.

Arterial blood gas (**Figure 10**) and hemodynamic parameters (**Figure 11**) were evaluated 7 days post-surgery to assess respiratory efficiency, metabolic balance, and cardiovascular stability in control, sepsis, probiotic, and sepsis + probiotic groups.

#### 3.5.1 Arterial blood gas parameters

PaO_2_ (Partial Pressure of Oxygen): Control group demonstrated normal arterial oxygenation of PaO_2_ 95 ± 5 mmHg. In the background, there is only minor sepsis-induced lung injury in the observation group, and there is a significant decrease of PaO_2_ in the sepsis group (60 ± 6 mmHg, p < 0.01). Probiotic treatment attenuated hypoxemia, resulting in an incrementally significant improvement in oxygenation (80 ± 5 mmHg in the probiotic group and 88 ± 6 mmHg in the sepsis + probiotic group, p < 0.05 vs sepsis).

Hypercarbia (PaCO_2_): Supranormal values of PaCO_2_ were shown in the sepsis group (50 ± 4 versus 38 ± 3 mmHg in controls, p < 0.01), indicating CO_2_ retention because of impaired ventilation. Probiotic administration decreased PaCO_2_ (mmHg) in the placebo (48 ± 3 mmHg), sepsis (40 ± 3 mmHg), and probiotic (42 ± 3 mmHg) groups (p < 0.05). This demonstrates a restoration of ventilatory function after probiotic administration.

Blood pH: The control group maintained a physiological pH (7.40 ± 0.03). There was a marked metabolic acidosis due to sepsis, with a pH of 7.25 ± 0.04 (p < 0.01). Probiotic therapy partially corrected acidosis (7.35 ± 0.03) in the probiotic (p < 0.05 vs sepsis), and normalized pH (7.25 ± 0.02, p < 0.05 vs sepsis) in the sepsis + probiotic.

HCO_3_^-^ (Bicarbonate: Serum bicarbonate levels were significantly reduced in the sepsis group (18 ± 2 mmol/L) compared to the control group (24 ± 2 mmol/L, p < 0.01), confirming metabolic acidosis. Probiotic-treated animals showed restored bicarbonate levels, with 22 ± 2 mmol/L in the probiotic group and 23 ± 2 mmol/L in the sepsis + probiotic group.

#### 3.5.2 Hemodynamic parameters

Heart rate (HR): The heart rate (320 ± 20 bpm) of the untreated group was normal. Sepsis was responsible for substantial tachycardia, where HR elevated to 420 ± 25 bpm (p < 0.01). Probiotic treatment significantly reduced this response, and the corresponding HR values were 360 ± 20 bpm in the probiotic group (probiotic vs no probiotic: P <0.0001) and 340 ± 18 bpm in the sepsis + probiotic group (sepsis + probiotic vs probiotic: P <0.0001), demonstrating improved cardiovascular stability.

Mean Arterial Pressure (MAP): MAP was stable in the control group (95 ± 6 mmHg) but significantly decreased in the sepsis group (65 ± 5 mmHg, p < 0.01), reflecting sepsis-induced hypotension. Probiotic treatment significantly improved MAP, with values of 80 ± 6 mmHg in the probiotic group and 88 ± 5 mmHg in the sepsis + probiotic group, approaching control levels.

Overall, sepsis caused profound disturbances in arterial blood gases and hemodynamic stability, characterized by hypoxemia, hypercapnia, metabolic acidosis, tachycardia, and hypotension. These abnormalities improved with probiotic treatment, with almost normal bulk respiratory and hemodynamic parameters observed in the sepsis + probiotic group, suggesting that probiotics protect against pulmonary and systemic dysfunction induced during sepsis.

## 4 Discussion

Our findings further strengthen the concept of the gut–lung axis as central to sepsis-induced lung injury and identify Lactobacillus rhamnosus GG as a stool microbiome-directed approach to improve lung and systemic outcomes in sepsis. As predicted by classic sepsis pathophysiology, we demonstrated significant pulmonary dysfunction (decreased tidal volume, minute ventilation, and lung compliance with reflex tachypnea), profound histologic lung injury (inflammation, alveolar damage, edema, hemorrhage), and generation of a pro-inflammatory cytokine response (TNF-α, IL-1β, IL-6) simultaneously with suppression of IL-10 in our CLP model. Similar other preclinical studies have shown that sepsis-induced dysregulated immunity, leading to endothelial injury, alveolar–capillary permeability, inflammatory infiltration, and impaired gas exchange, subsequently causes hypoxemia and ventilatory imbalance. Notably, many of these changes were restored by probiotic treatment in our study, suggesting that gut microbial interventions can modulate distal lung immunity and physiology through at least two mechanistic pathways.

One putative mechanism suggested by previous research is that sepsis results in a loss of intestinal epithelial barrier integrity, leading to enhanced intestinal permeability (“leaky gut”)  and the translocation of intestinal microbiota products (particularly LPS/endotoxin) into the systemic circulation (Bloebaum et al., 2023). This triggers TLR4-dependent pathways, thereby enhancing inflammation and lung injury. Supporting mechanism: reduction of alpha diversity and drive of dysbiosis with expansion of Proteobacteria (often elevated in inflammatory states) in sepsis microbiome data, which are linked to increased endotoxin load and barrier disruption (Rosendo-Silva et al., 2023). In contrast, both probiotic-treated groups displayed diversity restoration and a decrease in Proteobacteria, consistent with previous studies reporting that Lactobacillus strains promote tighter tight-junction complexes (occludin/claudins/ZO-1), decreased endotoxemia, and inhibition of downstream inflammatory signaling. This functional effect is reflected in improved PaO_2_, reduced PaCO_2_, and normalization of lung compliance, suggesting that hindgut-derived inflammatory spillover can be restrained to protect the lungs (Druszczynska et al., 2024).

Multiple previous studies designate gut–lung axis as an immune “relay” because gut dysbiosis induces systemic cytokine storm that attracts neutrophils and macrophages to the lung to trigger ALI/ARDS-like injury (Sun et al., 2025). Baseline levels of pro-inflammatory cytokines such as TNF-α, IL-1β, and IL-6 in healthy animals are typically low or near the detection limit of ELISA assays, while anti-inflammatory cytokines such as IL-10 are maintained at basal levels. Previous studies have shown that IL-6 and IL-1β levels in healthy controls are often very low or undetectable (<5 pg/mL), with significant elevations occurring only under inflammatory conditions. Similarly, TNF-α remains at low basal concentrations in non-diseased states. The cytokine values observed in the control group in the present study are consistent with these baselines physiological range. From a mechanistic standpoint, this implies that probiotics might redirect immune responses away from dysregulated NF-κB activation towards resolution pathways that underlie the histology improvements (lower injury scores, reduced hemorrhage/edema) that we observed (Niu et al., 2025). A slight increase in hemorrhage score observed in the probiotic group compared to the control group may be attributed to minor procedural or biological variability rather than a direct adverse effect of probiotic administration. Importantly, this variation was minimal and did not indicate pathological injury. These findings suggest that probiotic treatment does not induce lung damage but maintains tissue integrity close to baseline conditions.

In addition to composition, other studies emphasize microbial metabolites of gut microbiotas-most notably short-chain fatty acids (SCFAs) such as acetate, propionate, and butyrate-as important mediators of gut–lung communication (Ramos Meyers et al., 2022). By binding to G-protein-coupled receptors or inhibiting histone deacetylase, SCFAs decrease inflammatory signaling and increase epithelial barrier function and alveolar macrophage responsiveness (Luo et al., 2023). While we did not measure SCFAs directly, our observed replenishment of beneficial taxa (e.g., increased Lactobacillus/Bifidobacterium and restored Firmicutes–Bacteroidetes balance) is consistent with a metabolic transition towards healthy anti-inflammatory metabolite profiles (Xiao et al., 2022). Therefore, the observed improvements in gut microbiota composition following probiotic treatment may suggest, but do not confirm, a shift toward beneficial metabolite production. These interpretations should be considered cautiously and require further validation through targeted metabolomic analysis (Al-Husinat et al., 2024).

Dysbiosis in sepsis has also been shown in other studies to favor the expansion of opportunistic pathobionts (e.g., Enterobacteriaceae) that could exacerbate inflammation and secondary infections such as pneumonia (Stotts et al., 2023). This reported mechanism fits our genus-level trend (increased Escherichia/Enterococcus during sepsis, with probiotic-induced restoration of Lactobacillus). Lactobacilli are alleged to hinder pathogen proliferation through competitive exclusion, the production of organic acids/bacteriocins, and niche occupation. This mechanism is consistent with our reduced inflammatory load and improved blood gas parameters (Sulaiman et al., 2023). In contrast, this protective effect of microbial skin pathobionts appears at least partially dependent on oncostatin M production, which is associated with increased systemic inflammatory load and subsequent pulmonary injury.

After a period of intense inflammation (Margaria et al., 2022), early fibrotic signaling (TGF-β activation, extracellular matrix deposition) is observed in many sepsis/ARDS models. The sepsis group showed increased collagen deposition (fibrosis) as shown in our Masson Trichrome data, whereas the probiotic-treated groups showed significantly decreased collagen deposition, suggesting a reduction in early pro-fibrotic remodeling with probiotics. Similar studies indicate that probiotics indirectly inhibit TGF-β-mediated fibroproliferation and collagen deposition by decreasing IL-6/TNF-α levels and oxidative stress. Therefore, our findings are consistent with the literature, which suggests that early control of the initial inflammatory storm can improve downstream fibrotic remodeling and maintain compliance and long-term lung function (Savin et al., 2023).

Several reports indicate that gut-derived inflammation exacerbates sepsis-associated cardiovascular dysfunction by triggering vasoplegia and myocardial depression (Soranno et al., 2025). Rather, hypotension and tachycardia were observed in the sepsis group, but probiotics increased MAP and reduced tachycardia (Yang et al., 2025). Mechanistically, this may reflect reduced endotoxemia and cytokine burden, and better vascular tone, to limit systemic inflammatory vasodilation. This is a more general concept of the gut–organ axis, in which microbiome stabilization not only improves outcomes in the lungs but also likely contributes to systemic hemodynamic stability, further supporting oxygen delivery to and recovery of the respiratory system (Natalini et al., 2023). While this study provided valuable insights into the acute effects of probiotics on pulmonary function and inflammation, survival outcomes were not assessed. Future studies should aim to evaluate long-term survival and recovery to better understand the translational relevance of these findings. Although we hypothesize that the gut-lung axis mediates the observed benefits, it is also possible that probiotics exert systemic immune-modulating effects that contribute to the observed lung improvements, independent of microbiota changes. Further studies will be needed to disentangle these mechanisms.

### 4.1 Limitations

This study has certain limitations. Although sepsis was successfully induced using the widely accepted cecal ligation and puncture (CLP) model and confirmed through physiological, biochemical, and inflammatory markers, microbiological validation through bacterial culture was not performed. Future studies incorporating blood or peritoneal fluid cultures would provide more direct confirmation of infection and enhance translational relevance. The study did not include a non-surgical (naïve) control group. Although sham-operated animals were used to account for surgical stress, inclusion of a naïve control group would provide additional insight into the baseline physiological state and help further distinguish the independent effects of laparotomy. Future studies should incorporate both sham and naïve controls to improve experimental resolution. Additionally, microbiological culture studies were not performed, as the study primarily relied on 16S rRNA sequencing to comprehensively assess gut microbiota composition, which offers broader detection of microbial diversity compared to culture-based methods. Nevertheless, incorporation of blood or peritoneal cultures in future studies would enhance clinical validation of infection. Furthermore, key functional parameters such as short-chain fatty acids (SCFAs), advanced immune markers (e.g., TGF-β, IL-1ra), and immune cell phenotyping were not evaluated due to scope and feasibility constraints. The experimental duration was limited to 7 days, restricting assessment of long-term outcomes such as survival and chronic fibrosis. Future studies integrating metabolomics, extended observation periods, and additional control groups are warranted to provide deeper mechanistic insights and improve translational relevance.

### 4.2 Recommendations for the future

Our findings need to be validated in large-animal models and clinical trials to confirm the translatability of the probiotics in the sepsis context. Conversely, the integration of shotgun metagenomics, metabolomics, and transcriptomics would allow for deeper investigation of functional microbial pathways and host-microbiome interactions with respect to the gut-lung axis. More comparative studies with other probiotic strains, synbiotics, or postbiotics, and improved, better-optimized dosing regimens are needed to identify an efficacious treatment strategy. Longitudinal studies, however, would be clinically relevant for evaluating the sustainability of the survival benefit, lung remodeling, immune recovery, and the occurrence of secondary infections. Lastly, the synergism described herein could provide the foundation for microbiome-driven precision medicine in clinical practice; probiotics could be administered alongside standard sepsis treatments.

## 5 Conclusion

The research data provide strong experimental evidence that the gut–lung axis mediates pulmonary complications of sepsis and may inform the use of probiotics as a novel therapeutic strategy. Sepsis caused drastic adverse alterations in pulmonary mechanics, histological lung integrity, gut microbiota composition, arterial blood gases, and hemodynamic stability. Taken together, these changes represent the pathophysiological nature of severe sepsis, a process of cumulative or combined organ failure. The treatment with *Lactobacillus rhamnosus* GG significantly decreased sepsis-induced lung injury, as evidenced by improved tidal volume, minute ventilation, lung compliance, oxygenation, and carbon dioxide elimination, and fewer histopathological changes and collagen deposition in the lung. Treatment with probiotics inhibited the pulmonary cytokine cascade by downmodulating pro-inflammatory cytokines (TNF-α, IL-1β, IL-6) and upregulating the anti-inflammatory cytokine IL-10, thereby demonstrating immunologic rebalancing. Importantly, probiotics rebalanced the gut microbiota by restoring its diversity, restricting the proliferation of pathogenic bacteria, and reinstating beneficial populations, thereby mechanistically linking gut microbiota homeostasis with lung immunity. However, we demonstrate here for the first time that the gut microbiome can be manipulated to stabilize systemic physiology and reduce multi-organ dysfunction in sepsis, as evidenced by improved arterial blood gases and hemodynamic parameters. The findings of this study suggest that probiotic supplementation may improve pulmonary function, modulate inflammatory responses, and restore gut microbiota composition in a CLP-induced sepsis model. These findings support the potential involvement of the gut–lung axis in sepsis-associated pulmonary dysfunction. However, given the preclinical nature of the study, the use of a single probiotic strain, and the absence of detailed mechanistic validation, further studies are required to confirm these observations and to explore their translational relevance in clinical settings.

## Data Availability

The original contributions presented in the study are included in the article/supplementary material. Further inquiries can be directed to the corresponding author.
